# Comment on “report of 5 novel mutations of the α-L-iduronidase gene and comparison of Korean mutations in relation with those of Japan or China in patients with mucopolysaccharidosis I”

**DOI:** 10.1186/s12881-018-0697-3

**Published:** 2018-10-04

**Authors:** Edina Poletto, Ursula Matte, Guilherme Baldo

**Affiliations:** 1 Postgraduate Program in Genetics and Molecular Biology, Universidade Federal do Rio Grande do Sul, Porto Alegre, Brazil; 2 Gene Therapy Center, Hospital de Clínicas de Porto Alegre, Ramiro Barcelos, 2350, Porto Alegre, RS 90035-903 Brazil

**Keywords:** IDUA, 704ins5, C.613_617dupTGCTC

## Abstract

In this comment, we highlight that the *IDUA* pathogenic variants 704ins5 and c.613_617dupTGCTC are the same, but have different names depending on the nomenclature guideline used. Therefore, the frequency of this variant is 17.6% of alleles in Korean patients. This commentary stresses the importance of proper variant annotation and the use of guidelines when describing or reviewing mutations.

## Background

Ever since the *IDUA (α-L-iduronidase)* gene was first described [1], numerous pathogenic variants have been reported. However, the nomenclature guideline for human sequence variants has been updated over the years, and well known variants acquired new names. Since mutational profile studies frequently highlight differences in the allele frequencies in order to analyse and compare different populations, it is important to keep a sharp eye in the current guideline nomenclature, as it can influence the frequencies obtained and the conclusions of the study.

## Main text

### 704ins5/c.613_617dupTGCTC

In a paper published last year by Kwak and colleagues, “Report of 5 novel mutations of the α-L-iduronidase gene and comparison of Korean mutations in relation with those of Japan or China in patients with Mucopolysaccharidosis I”, a misunderstanding has been identified regarding the *IDUA* gene mutations 704ins5 and c.613_617dupTGCTC. The authors have considered them as two different mutations found in Korean patients with Mucopolysaccharidosis type I (MPS I), with frequencies estimated in approximately 12% and 6% of analysed alleles, respectively. We would like to point out that these mutations are the same and have different nomenclatures due to updates in nomenclature guidelines, which switched 704ins5 to c.613_617dupTGCTC, as appears in the Human Gene Mutation Database (HGMD) [2].

**Fig. 1:**
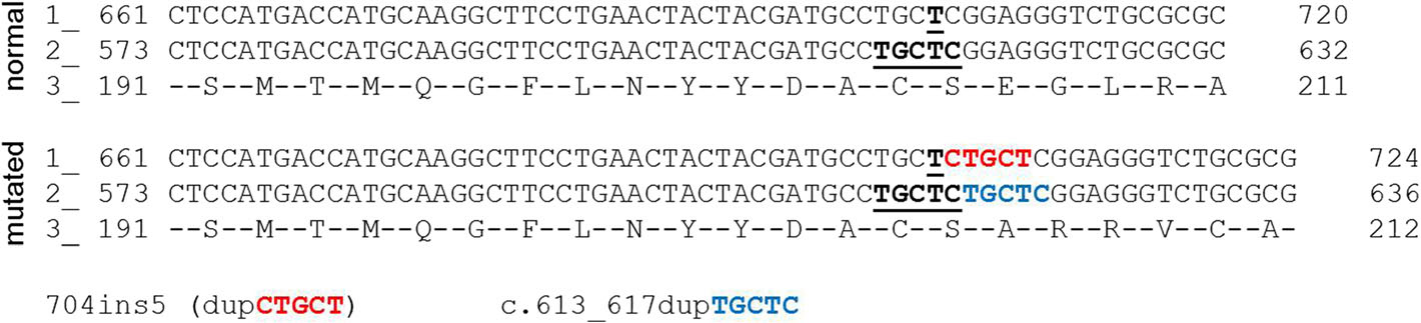
Part of IDUA cDNA sequence obtained from Ensembl (www.ensembl.org, RefSeq NM_000203) highlighting mutation 704ins5/ c.613_617dupTGCTC. 1- Number refers to the nucleotide position considering number 1 as first nucleotide of cDNA (c.-88). 2- Number refers to the nucleotide position considering number 1 as the A from the first ATG (c.1). 3- Number refers to amino acid position considering number 1 as the first Methionine (p.M1). Black bold: positions 704 and 613_617 in line 1 and 2, respectively. Red: nucleotides inserted according to Yamagishi et al., 1996, when first describing mutation 704ins5. Blue: nucleotides duplicated in mutation c.613_617dupTGCTC. Both result in same nucleotide alteration, but were presented with different nomenclatures

The mutation 704ins5 was first described in 1996 by Yamagishi and colleagues. In the original paper, it says the mutation was a duplication of a short sequence (CTGCT) and the position 704 was obtained considering the nucleotide 1 as the first of the cDNA sequence, not taking the translation initiation codon (ATG) into account. According to the current guidelines from the Human Genome Variation Society (HGVS - http://varnomen.hgvs.org), the nomenclature must follow prioritisation: duplication before insertion, most 3′ possible and cDNA considering position “c.1” as the A of the ATG start codon, and the the upstream regions considered as “c.-”. Therefore, 704ins5 becomes c.613_617dupTGCTC, as illustrated in Fig. 1.

## Conclusion

In conclusion, since 704ins5 and c.613_617dupTGCTC are the same variant, its frequency in Korean patients is 17.6% of mutated alleles, being the second most frequent variant in MPS I patients in this population [3, 4], similar to what was observed in Japan, with 18.4% of alleles [5]. This commentary stresses the need for everyone involved in variant description, including authors, reviewers and readers alike, to bear in mind the importance of variant annotation and to use the most up-to-date guidelines.

## Abbreviations

HGMD: Human Gene Mutation Database
HGVS: Human Genome Variation Society
IDUA: α-L-Iduronidase
MPS I: Mucopolysaccharidosis type I
